# Longitudinal Impact of the Project PATHS on Adolescent Risk Behavior: What Happened after Five Years?

**DOI:** 10.1100/2012/316029

**Published:** 2012-01-29

**Authors:** Daniel T. L. Shek, Lu Yu

**Affiliations:** ^1^Department of Applied Social Sciences, The Hong Kong Polytechnic University, Hunghom, Hong Kong; ^2^Public Policy Research Institute, The Hong Kong Polytechnic University, Hong Kong; ^3^Department of Social Work, East China Normal University, Shanghai 200241, China; ^4^Kiang Wu Nursing College of Macau, Macau, China; ^5^Division of Adolescent Medicine, Department of Pediatrics, Kentucky Children's Hospital, University of Kentucky College of Medicine, Lexington, KY 40506, USA

## Abstract

The present study investigated the longitudinal impact of the Project PATHS, a large-scale curriculum-based positive youth development program in Hong Kong, on the development of adolescents' risk behavior over a period of five years. Using a longitudinal randomized controlled design, eight waves of data were collected from 19 experimental schools in which students participated in the Project PATHS (*N* = 2,850 at Wave 8) and 24 control schools without joining the Project PATHS (*N* = 3,640 at Wave 8). At each wave, students responded to measures assessing their current risk behaviors, including delinquency, use of different types of drug, and their intentions of participating in risk behaviors in the future. Results demonstrated that adolescents receiving the program exhibited significantly slower increases in delinquent behaviors and substance use as compared to the control participants. During two years after the completion of the program, differences in youth risk behaviors in the two groups still existed. These results suggest that the Project PATHS has long-term effect in preventing adolescent problem behavior through promoting positive youth development.

## 1. Introduction

Adolescent risk behavior, such as substance use, delinquency, risky sexual behavior, violence, and school failure, increases the likelihood of adversity in many life domains: physical health, psychological well-being, and psychosocial development, as well as the stability of the society. In the past decades, research has shown that adolescent risk behavior is a growing concern. The urgent need of developing effective evidence-based programs, strategies, and policies to prevent youth risk behaviors has been repeatedly emphasized by researchers, practitioners, and policy makers across the world. Accordingly, numerous studies have been conducted to identify specific risk and protective factors for the unfolding of risk behaviors during adolescence, based on which a proliferation of prevention programs have been developed and implemented worldwide [1, 2].

In recent years, a focus on youth strengths, developmental assets, and positive attributes in adolescents has been increasingly advocated and incorporated into the risk behavior prevention models, called the positive youth development approach. While there are many different positive youth development models, the common and basic principles of this approach include “problem free” is not “fully prepared” for adolescents; the presence of positive attributes, instead of the absence of problems, denotes successful youth development; both “problem free” and “fully prepared” can be achieved by promoting positive competences and characters in youth. For example, the Search Institute has proposed a developmental assets model in which forty youth developmental assets (e.g., internal and external qualities) are highlighted for healthy development during adolescence [3]. In a comprehensive literature review, Catalano summarized 15 positive youth development constructs from 25 successful positive youth development programs in the United States [1]. Based on rigorously designed evaluative studies, researchers have reported encouraging findings that support the effects of programs adopting the positive youth development approach in decreasing adolescent risk behaviors and promoting positive developmental outcomes [4–6].

While most positive youth development programs are developed and implemented in the West, the Project PATHS is probably the largest youth enhancement program in Asian countries, which was initiated by the Hong Kong Jockey Club Charities Trust. The word “PATHS” denotes Positive Adolescent Training through Holistic Social Programmes. In 2005, Shek and researchers from five universities in Hong Kong designed the project with the aim to promote positive development among Hong Kong adolescents and reduce their risk/problem behaviors [7, 8]. There are two tiers of programs in the project. Tier 1 program is a universal curriculum-based program developed upon 15 positive youth development constructs proposed by Catalano and colleagues [1], including bonding, resilience, social competence, recognition of positive behavior, emotional competence, cognitive competence, behavioral competence, moral competence, self-determination, self-efficacy, clear and positive identity, beliefs in the future, prosocial involvement, prosocial norms, and thriving. Tier 2 program adopts a selective approach targeting at about one fifth of students who have greater psychosocial needs. The project has been implemented in roughly half of the total number of secondary schools in Hong Kong for consecutively five years.

To determine whether and the extent to which a positive youth development program can effectively achieve its intended outcomes, it is critical to conduct rigorously designed evaluation studies to evaluate the program. Since its inception, the Project PATHS has been evaluated by various evaluative strategies, including subjective outcome evaluation by different stakeholders (participants and program implementers), objective outcome evaluation (quantitative methods and qualitative methods), process evaluation, classroom observation, and repertory grid tests. Available evaluation findings consistently show that the project is effective in promoting positive youth development among Chinese adolescents in Hong Kong, and that both program implementers and participants expressed positive views towards the program. With particular regard to objective outcome evaluation, Shek and Sun reported that program participants showed desirable changes in many positive youth development domains and displayed less problem behaviors than did participants in the control group [9].

One of the most prominent evaluative strategies used in the Project PATHS is the employment of a longitudinal randomized control group trial to trace the developmental trajectory based on different risk behavior and positive developmental outcome indicators in students who participated in the project and a group of control students. As suggested by Sibbald and Roland [10], randomized control trial is the most stringent way of determining whether a cause-effect relation exists between the intervention and the outcome. The trial for evaluating the Project PATHS started in the academic year of 2006, with 19 experimental schools and 25 control schools participated in the study. The first two waves of data collected in the full implementation phase showed that participants in the experimental group exhibited greater improvements in different positive youth development constructs at posttest than did the control group students [11]. Based on the first four waves of data in the trial, Shek and Sun reported that students who participated in the program had significantly better positive outcomes in terms of psychosocial competency, academic and school behavior, and global positive youth development while exhibited lower levels of delinquent behaviors as compared to students in the control group [12]. After six waves of data collection, a more advanced statistical method, Hierarchical Linear Modeling (HLM), has been used to investigate program impacts on participants over time, which enables researchers to estimate individual growth curves of each behavioral indicator in a more precise way, as compared to the traditional analyses of covariance. Using linear mixed models via SPSS, significant effects of the project on positive youth development and risk behaviors based on the six waves of data have been reported. Participants in the experimental schools not only displayed better positive youth development outcomes but also showed slower increases in delinquent behaviors than did the control participants over the three years of program implementation [13, 14].

A good positive youth development program should have both short-term and long-term effects in its participants. While there are positive findings supporting the immediate effects of the Project PATHS in preventing risk behaviors, it is unknown whether these effects could last after completion of the program. Therefore, to evaluate the long-term effects of the Project PATHS on the developmental trajectory of youth risk behaviors, the randomized control group trial continued to collect data from the participants after the three-year program had finished. Until now, a total of eight waves of data have been collected with the last two waves collected in one and two years after the completion of the project, respectively. The purpose of the present study is to examine the effect of the Project PATHS on preventing adolescent risk behaviors based on the eight waves of data collected during five consecutive years. Consistent with previous studies, linear mixed-effect modeling via SPSS was employed for data analyses.

## 2. Method

### 2.1. Participants and Procedures

The detailed procedure and criteria of recruiting participants for the randomized controlled group trial were described in our previous papers [13–15]. In brief, 24 experimental schools and 24 control schools were randomly selected in Year 1, with one experimental school dropped out after the first year implementation of the project. Therefore, Wave 1 and Wave 2 data were collected from Secondary 1 students in 23 experimental schools and 24 control schools. In Year 2, Wave 3 and Wave 4 data were collected from the same cohort who upgraded to Secondary 2, with 20 experimental schools (i.e., three schools withdrew after Wave 2) and 24 control schools participated in. In Year 3, Wave 5 and Wave 6 data were collected from the same cohort in Secondary 3 at that time, including 19 experimental schools (i.e., one experimental school dropped out after Wave 4) and 24 control schools. In Year 4, that is, one year after the completion of the Project PATHS, Wave 7 data were collected from the same cohort who entered to Secondary 4, including 19 experimental schools and 24 control schools. In Year 5, Wave 8 data were collected from the same cohort of students who were in Secondary 5, with 19 experimental schools and 24 control schools attended the study two years after the completion of the program.  Table 1 shows the number of completed questionnaires collected in each wave.

At each measurement occasion, the purposes of the study were introduced and confidentiality of the data collected was repeatedly ensured to all participants in attendance on the days of survey. Parental and student consent forms had been obtained before data collection. Participants responded to the questionnaires in a self-administration format in classroom settings. A trained research assistant was present throughout the administration process.

### 2.2. Instruments

Consistent with procedures employed in previous studies, participants were required to respond to a composite questionnaire that comprises different measures of youth development constructs and problem behaviors. The internal consistency of each measure used in this study at different waves is presented in Table 2.

#### 2.2.1. Chinese Positive Youth Development Scale (CPYDS)

The CPYDS consists of 15 subscales which are listed as follows.

1. Bonding Subscale (six items).
2. Resilience Subscale (six items).
3. Social Competence Subscale (seven items).
4. Emotional Competence Subscale (six items).
5. Cognitive Competence Subscale (six items).
6. Behavioral Competence Subscale (modified five items).
7. Moral Competence Subscale (six items).
8. Self-Determination Subscale (five items).
9. Self-Efficacy Subscale (modified two items).
10. Beliefs in the Future Subscale (modified three items).
11. Clear and Positive Identity Subscale (seven items).
12. Spirituality Subscale (seven items).
13. Prosocial Involvement Subscale (five items).
14. Prosocial Norms Subscale (five items).
15. Recognition for Positive Behavior Subscale (four items).

It should be noted that although the administered questionnaire includes the CPYDQ, findings based on CPYDQ, and its subscales were reported elsewhere [16]. The present paper only focused on the development of problem behaviors among students in the experimental schools and the controlled schools, including delinquent behaviors, substance abuse, and intentions of engaging in problem behaviors in the future.

#### 2.2.2. Delinquency Scale

This scale comprises 12 items that assess the frequency of delinquent behavior of the participants in the past year, including stealing, cheating, truancy, running away from home, damaging others' properties, assault, having sexual relationship with others, gang fighting, speak foul language, staying away from home with parental consent, strong arm others, and break in others' places [17]. Respondents rated the frequency of these behaviors in the past half a year on a six-point Likert-scale (0 = never, 1 = 1-2 times; 2 = 3-4 times; 3 = 5-6 times; 4 = 7-8 times; 5 = 9-10 times; 6 = more than 10 times). Both the scale score and each item score were used in the analyses. The Cronbach's alpha of the delinquency scale was 0.80 and 0.76. at Wave 7 and Wave 8, respectively.

#### 2.2.3. Substance Use Scale

Eight items were used to assess the participants' frequency of using different types of substance in the past half a year, including alcohol, tobacco, ketamine, cannabis, cough mixture, organic solvent, ecstasy, and heroin. Participants rated their occurrence of these behaviors on a six-point Likert-scale (0 = never; 1 = 1-2 times; 2 = 3–5 times; 3 = more than 5 times; 4 = several times a month; 5 = several times a week; 6 = everyday). In this study, in addition to the scale score of substance use (i.e., mean score of the eight items) and each item score, several composite scores were created for analyses including CAS (tobacco and alcohol use), IPS (use of illegal drugs: ketamine, cannabis, ecstasy, and heroin), and LPS (use of legal drugs: cough mixture, organic solvent). Scores of CAS, IPS, and LPS were calculated by averaging the relevant item scores. The internal consistency of this scale was 0.78 at Wave 7 and 0.72 at Wave 8.

#### 2.2.4. Problem Behavior Intention Scale

Five items were used to assess the participants' behavioral intention to engage in problem behavior including drinking alcohol, smoking, taking drugs (such as Ketamine, cannabis or ecstasy), having sex with others, and gambling [18]. Respondents were asked to rate the likelihood that they may engage in these problem behaviors in the next two years on a four-point Likert-scale, with “1” representing for “never,” “2” for “not likely,” “3” for “likely,” and “4” for “definitely.” The program behavior intention scale score was used in the analyses and the internal consistency for the scale at Wave 7 and Wave 8 was 0.76 and 0.73, respectively.

### 2.3. Data Analytic Plan

In the present study, we adopted the individual growth curve modeling (IGC) approach as recommended by Shek and Ma [19], to the analysis of adolescents' individual change in problem behaviors over time and the examination of the longitudinal effects of the Project PATHS on the developmental trajectories of different youth problem behaviors. Both composite indicators (i.e., scale scores of delinquency, substance abuse, and problem behavior intention) and individual item scores were treated as dependent variables.

The use of IGC in studying longitudinal data has been detailed in many articles [20]. In a nutshell, longitudinal data are considered as a two-level hierarchical model in which time is nested within individuals [21, 22]. The Level 1 model refers to the intraindividual change model that models the variation within individual over time and estimates the average within-person initial status and the average rate of change over time. In other words, the outcome variable is represented as simply the function of time without any other predictors involved. The Level 2 model captures whether the rate of change varies across individuals in a systematic way. The growth parameters estimated in the Level 1 model serve as the outcome variables in the Level 2 model which are further predicted by various interindividual variables. At this step, different explanatory variables such as “participation in the program” can be included to analyze their effects on the interindividual variation of outcome variables.

More information about how to formulate and interpret the model can be seen in the papers by Shek and Ma [19]. In simple words, the longitudinal effects of the program on youth problem behavior were tested by examining whether “participating in the Project PATHS” was predictive of students' growth parameters (i.e., initial status, linear change, quadratic change, and cubic change) in different problem behavior indicators across time, with the effects of gender and initial age being controlled. In the IGC model, the intercept (i.e., initial status) and linear slope were allowed to vary across individuals.

First, a dummy/dichotomous variable was created (i.e., *group—*experimental group versus control group) as a major predictor. Participants in the control group were coded as −1 and those in the experimental group as 1. Two covariates (i.e., gender and initial age) were included when examining the predictive program effects on the outcome variables. *Gender* was coded as −1 = male and 1 = female. Following Shek and Ma's method [13], continuous variables were grand-mean centered in order to simplify the interpretation of the results [16]. In this study, the mean age was 12. *Initial age* was then centered by subtracting the mean age, and therefore, the centered initial age was generated.

To facilitate the interpretation of the significant interaction effects (between time variables and the program), the prototypical trajectories were plotted as suggested by Singer and Willett [23] to illustrate the effect of treatment on the rate of change across time. The step in creating prototypical plots is generally identical to the method of plotting graphs in regression [24]. For each outcome variable, a linear mixed model (LMM) via SPSS with maximum likelihood estimation was conducted. As the focus was on the entire model (both fixed and random effects), maximum likelihood (ML) method was used [25]. The procedures for analyzing longitudinal data via SPSS can be seen in Shek and Ma's paper [19].

## 3. Results

With schools being the units of analysis, results indicated that the 19 experimental schools and 24 control schools did not differ in school characteristics in terms of banding (i.e., categorizing based on students academic competence), geographic district, religious affiliation, sex ratio of the students, and source of funding. At the individual level, preliminary analyses showed that there were no statistically significant differences between the two groups in all sociodemographic background characteristics of the students (*P* > 0.05), but age. The mean age of the control group was higher than that of the experimental group. In other words, the background characteristics of the experimental schools and control schools were highly comparative at Wave 1.

Table 3 presents the IGC findings based on different problem behavior indicators. Results showed that there were significant interactions of group and slopes for substance use (scale score), delinquency (scale score), and problem behavior intention (scale score). Using individual item score as dependent variables, significant interactions of group and slopes were found in the use of ketamine, cannabis, organic solvent, ecstasy, and heroin, having sexual behavior, violence, stay outside home overnight, and trespasses.

### 3.1. Delinquency

**Figure 1:**
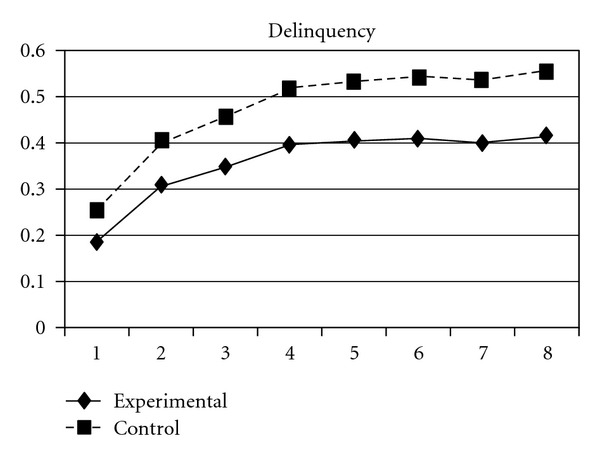
Growth trajectories of the experimental group (all participants) and control group using delinquency scale score as the outcome indicator.

**Figure 2:**
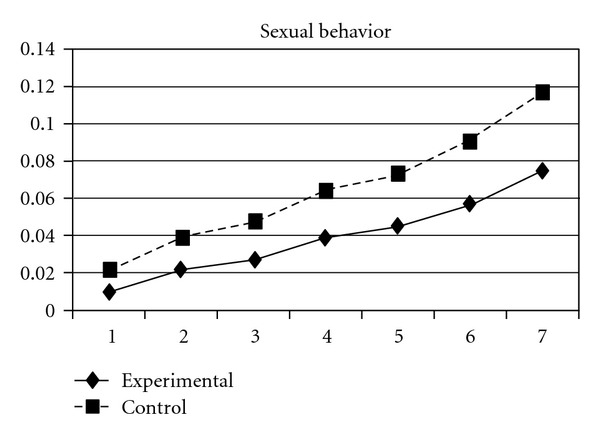
Growth trajectories of the experimental group (all participants) and control group using the item score of having sexual behavior with others as the outcome indicator.

**Figure 3:**
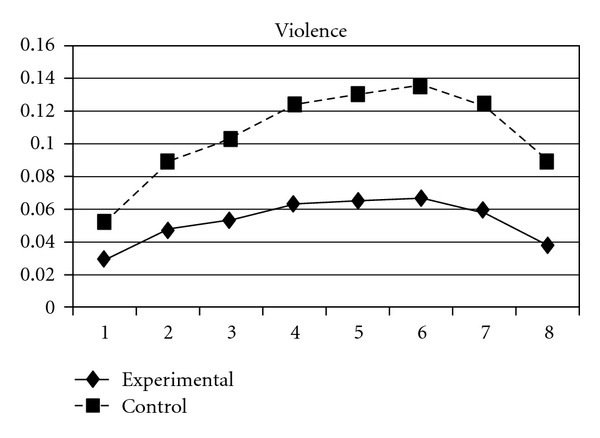
Growth trajectories of the experimental group (all participants) and control group using the item score of violence as the outcome indicator.

**Figure 4:**
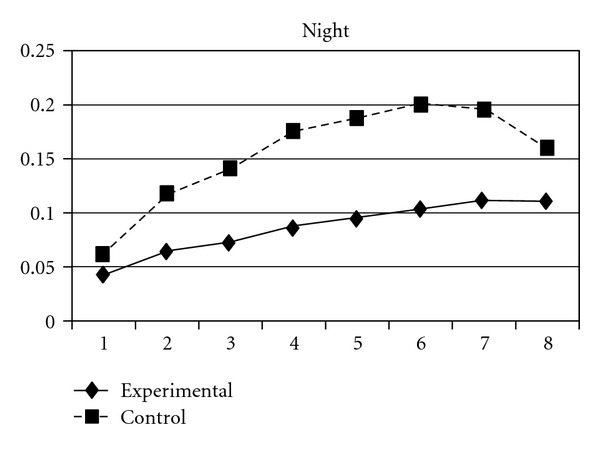
Growth trajectories of the experimental group (all participants) and control group using the item score of stay outside home over night as the outcome indicator.

**Figure 5:**
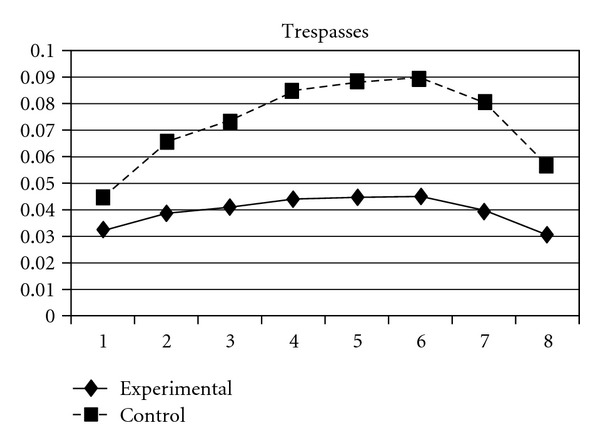
Growth trajectories of the experimental group (all participants) and control group using the item score of trespasses as the outcome indicator.

For both the experimental group and the control group, delinquent behaviors increased over time, following a cubic developmental trend. Group was a significant predictor of the initial status and linear slope (*P* = 0.03) but was unrelated to quadratic and cubic slopes (*P* > 0.05). Group differences in the initial status (**β** = −.03, SE =  .01, *P* < 0.001) and linear slope (**β** = −.03, SE =  .01, *P* < 0.05) indicate that the experiment group scored lower at the beginning of the study and had a slower rate of increase than the control group. As can be seen in Figure 1, these results suggest that, across the six waves of data collection, the experimental group consistently exhibited lower levels of delinquent behaviors compared to the control group. Using individual delinquent behavior as the dependent variable, significant group effects over time were detected in sexual (i.e., having sexual behaviors with others), violence, night (staying outside home overnight), and trespasses. First, the group and linear slope interaction was significant for sexual behavior (**β** = −.004, SE =  .002, *P* < 0.05), indicating that the experimental group had a slower rate of increase than did the control group (Figure 2). Second, violence, night, and trespasses all followed a quadratic developmental model, in which group effects were significant for both linear and quadratic slopes. As can be seen in Figures 3, 4, and 5, the experimental group increased slower than did the control group in the three forms of delinquent behaviors, but after Wave 6 (when the program was completed and the students entered into high secondary school), the control group showed a faster deceleration in delinquent behaviors as compared to the experimental group. 

### 3.2. Substance Use

**Figure 6:**
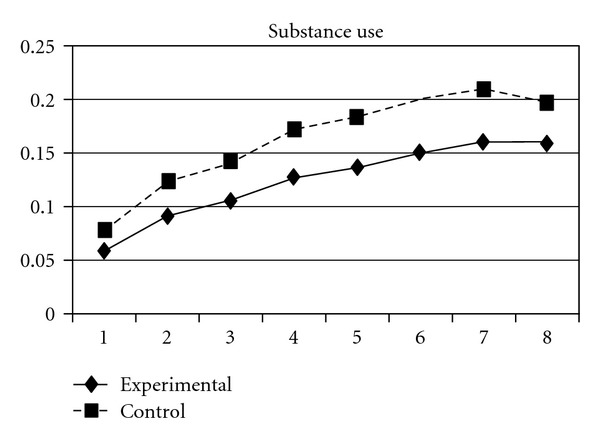
Growth trajectories of the experimental group (all participants) and control group using substance use scale score as the outcome indicator.

The development of substance abuse behavior in the present sample also followed a quadratic trajectory, with participants' use of substance increased with time. Significant effects of group were found in both the linear (**β** = −.01, SE =  .004, *P* < 0.001) and quadratic slopes (**β** =  .001, SE =  .001, *P* < 0.05) of substance use but not in the initial status. This means that while the experimental group and the control group did not differ in their initial status of substance abuse, the control group displayed a faster rate of increase and a slower rate of deceleration (quadratic slope) than did the experimental group. The developmental curves of substance use behaviors for the two groups can be seen Figure 6. 

**Figure 7:**
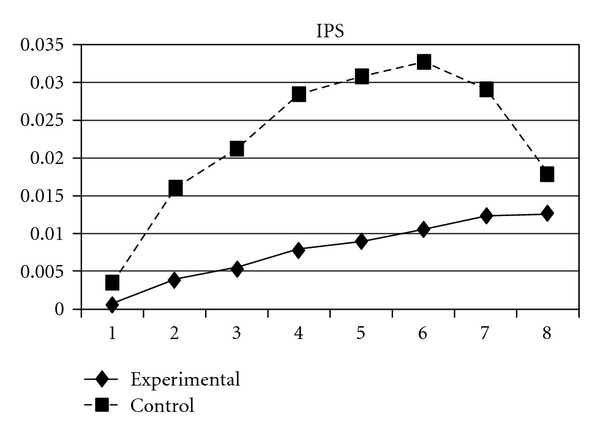
Growth trajectories of the experimental group (all participants) and control group using the composite score of illegal drug use as the outcome indicator.

**Figure 8:**
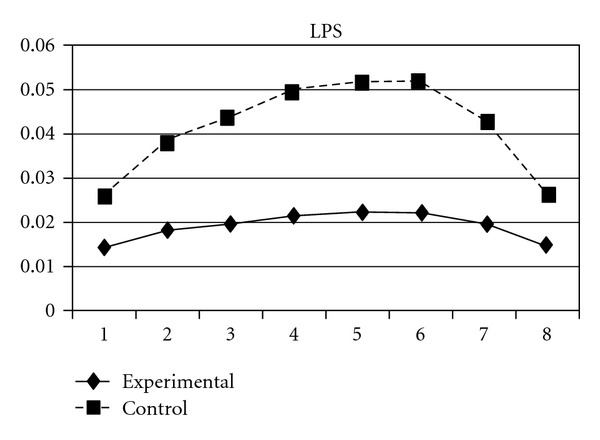
Growth trajectories of the experimental group (all participants) and control group using the composite score of legal drug use as the outcome indicator.

**Figure 9:**
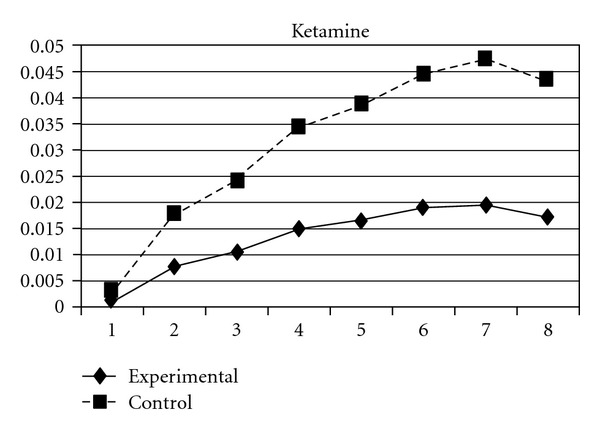
Growth trajectories of the experimental group (all participants) and control group using the item score of ketamine use as the outcome indicator.

**Figure 10:**
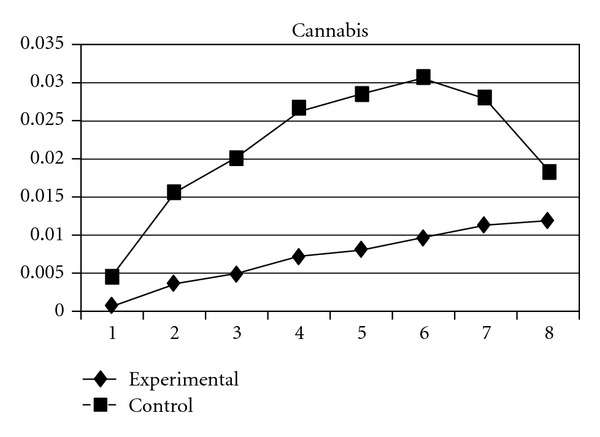
Growth trajectories of the experimental group (all participants) and control group using the item score of cannabis use as the outcome indicator.

**Figure 11:**
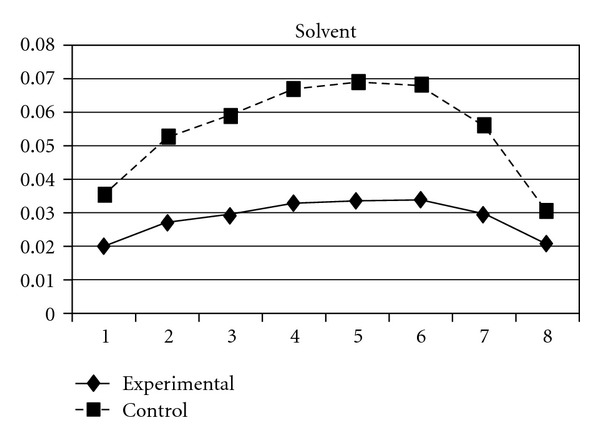
Growth trajectories of the experimental group (all participants) and control group using the item score of solvent use as the outcome indicator.

**Figure 12:**
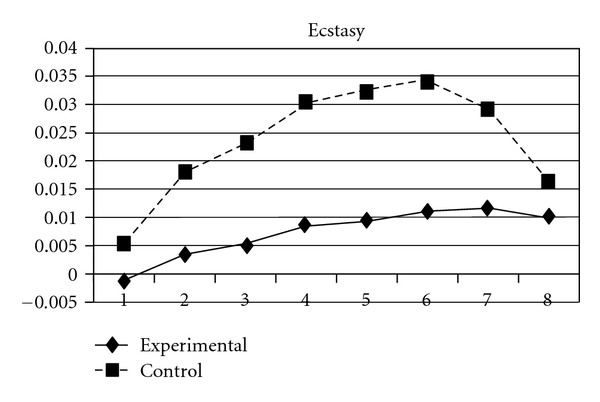
Growth trajectories of the experimental group (all participants) and control group using the item score of ecstasy use as the outcome indicator.

**Figure 13:**
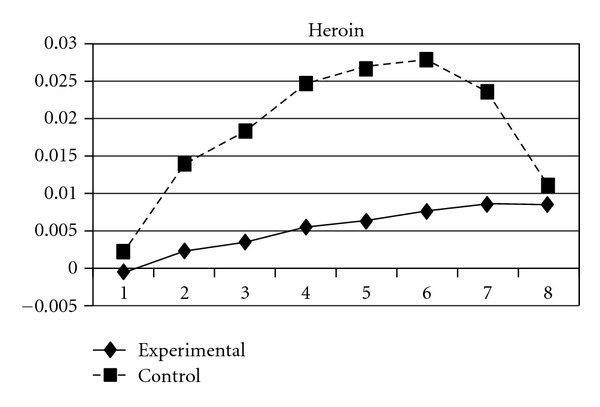
Growth trajectories of the experimental group (all participants) and control group using the item score of heroin use as the outcome indicator.

As mentioned earlier, because adolescents' use of different types of substances may have different developmental trajectories, other three composite scores, CAS (tobacco and alcohol use), IPS (illegal drug use), and LPS (legal drug use), were created and included in the analyses. The results showed that while the group effect was nonsignificant on CAS, for IPS and LPS, the experimental group and the control group significantly differ in the linear slopes (**β** = −.01, SE =  .004, *P* < 0.05 for IPS; **β** = −.01, SE =  .004, *P* < 0.05 for LPS) and quadratic slopes (**β** =  .001, SE =  .001, *P* < 0.05 for IPS; **β** =  .002, SE =  .001, *P* < 0.05 for LPS) of both illegal and legal drug use. As shown in Figures 7 and 8, for both IPS and LPS, the control group first showed a significantly faster increase in drug use and then had a faster deceleration than did the experimental group, especially after wave 6. Similar developmental curves can also be observed in individual drug use, including ketamine, cannabis, solvent, ecstasy, and heroin use (see Figures 9, 10, 11, 12, and 13). 

### 3.3. Problem Behavior Intention

**Figure 14:**
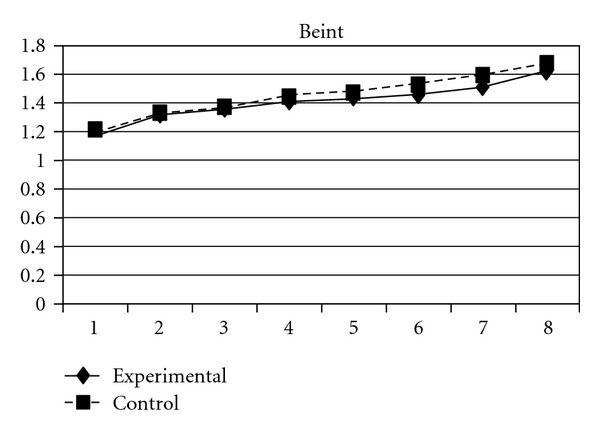
Growth trajectories of the experimental group (all participants) and control group using the scale score of problem behavior intention as the outcome indicator.

Students' intentions of engaging in problem behaviors in the future followed a cubic developmental trajectory. Significant group difference was found on the quadratic (**β** = −.01, SE =  .01, *P* < 0.05) and cubic slopes (**β** =  .002, SE =  .001, *P* < 0.01), but not the linear slope. This means that although the initial linear increase in students' problem behavior intention among the two groups did not differ significantly, the control group displayed a slower deceleration and a faster cubic development than did the experimental group. As can be seen in Figure 14, the experimental group consistently showed lower levels of problem behavior intention as compared to the control group across the 8 waves. 

 To provide further support for the effectiveness of the program, participants in the experimental group who perceived the program as beneficial to their development were selected and compared to the control participants. Significant group effects on growth parameters were found again in both composite indicators (delinquency, problem behavior intention, CAS, IPS, and LPS) and different item scores of problem behaviors (including use of tobacco, alcohol, solvent, ecstasy, and heroin, damaging other's property, violence, stay outside home overnight, and trespasses), as shown in Table 4. The patterns of group effects on changes in these behaviors were basically consistent with the patterns found in comparing all participants in the experimental group and the control group reported earlier. In addition to variables showing significant group effects in previous analyses, other four indicators were found to differ in their growth parameters between the experimental group and the control group. 

**Figure 15:**
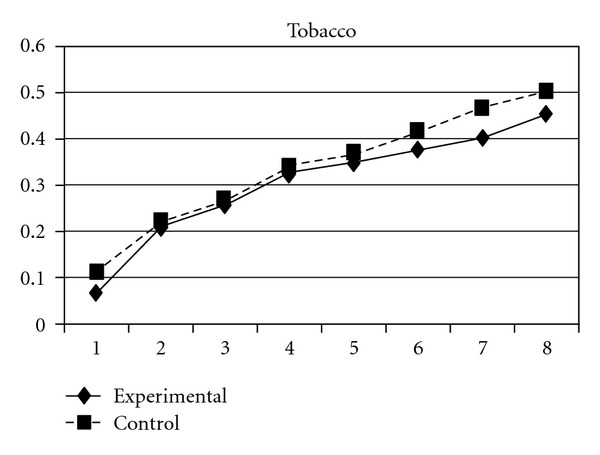
Growth trajectories of the experimental group (participants who perceived the program as effective) and control group using the scale score of problem behavior intention as the outcome indicator.

**Figure 16:**
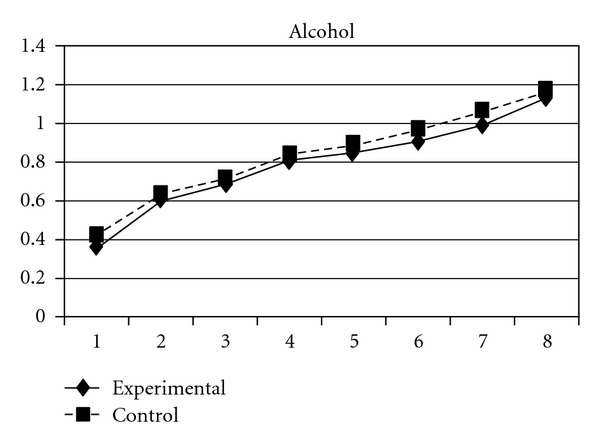
Growth trajectories of the experimental group (participants who perceived the program as effective) and control group using the scale score of problem behavior intention as the outcome indicator.

**Figure 17:**
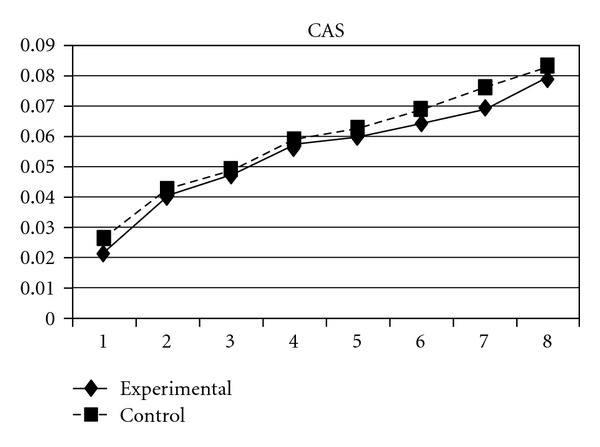
Growth trajectories of the experimental group (participants who perceived the program as effective) and control group using the composite score of tobacco use and alcohol use as the outcome indicator.

Three indicators of substance use, tobacco use, alcohol use, and their composite score (CAS), showed different developmental trends in the two groups. Group effects were significant on linear slopes (**β** =  .04, SE =  .02, *P* < 0.05 for tobacco use; **β** =  .04, SE =  .02, *P* < 0.05 for alcohol use; **β** = −.04, SE =  .02, *P* < 0.01 for CAS), quadratic slopes (**β** = −.02, SE =  .01, *P* < 0.05 for tobacco use; **β** = −.02, SE =  .01, *P* < 0.05 for alcohol use; **β** = −.02, SE =  .01, *P* < 0.01 for CAS), and cubic slopes (**β** =  .003, SE =  .001, *P* < 0.05 for tobacco use; **β** =  .003, SE =  .001, *P* < 0.05 for alcohol use; **β** =  .003, SE =  .001, *P* < 0.01 for CAS). This indicates that the initial rates of increase in the three indicators were more rapidly in the experimental group than in the control group; however, the experimental group showed a faster deceleration and cubic development in the rate of increase than did the control group. Figures 15, 16, and 17 depict the developmental curves of the three indicators. 

**Figure 18:**
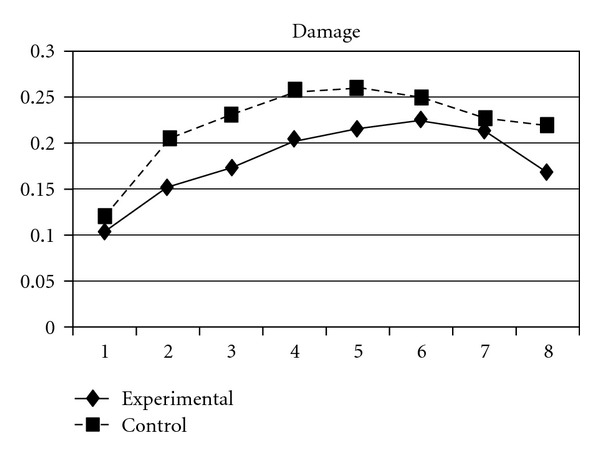
Growth trajectories of the experimental group (participants who perceived the program as effective) and control group using the item score of damaging other's property as the outcome indicator.

 Significant group effects were also found on the growth parameters of one extra indicator of delinquent behavior: damage (damaging other's property). The experimental group showed a slower rate of increase (**β** = −.04, SE =  .02, *P* < 0.05), a slower deceleration (**β** =  .02, SE =  .01, *P* < 0.01), and finally a faster cubic deceleration (**β** = −.003, SE =  .001, *P* < 0.01) than did the control group. The growth curves for damage in the two groups are pictured in Figure 18. 

 Growth trajectories of other problematic behaviors in the two groups were not plotted in this paper due to the limited space. Relevant information is available for readers upon request.

## 4. Discussion 

The purpose of this paper is to report objective outcome evaluation findings over a period of five years regarding the effectiveness of a positive youth development program (Project PATHS) in Hong Kong using individual growth curve modeling technique. This is the first known scientific study that adopted a randomized group trial design using data spanning over five years to evaluate a positive youth development program based on a curricular approach in the context of Chinese culture. 

There are several benefits of the rigorous methodology used in the present study. The first is related to the utilization of a group randomized control trial. Researchers have pointed out three major errors that often affect the evaluative results in program evaluation [26]: bias (i.e., the deviation of results from the truth caused by systematic error in the research methodology), confounding factors (characteristics of the subject that are related to the intervention outcomes), and chance (random errors that may cause the link between an intervention and an outcome). However, by using a well-designed randomized control trial [27], “these errors can all be effectively reduced or designed out.” (page 164). Second, the present study has a very large sample, which is considered the most important strategy to reduce random error [10]. Third, longitudinal data were collected not only within the period of program implementation but also after the completion of the program. Based on the eight waves of data collected, individual growth curve modeling was used to compare the developmental trajectories of adolescent risk behaviors in the experimental group and in the control group, by which both short-term and long-term effects of the program were detected. 

The results generally showed that compared with participants in the control group, participants in the experimental schools performed better in different indicators of adolescent risk behavior. First, the findings revealed that experimental participants displayed lower level of delinquent behavior and the acceleration rate was slower than that of the control participants. Second, with reference to substance abuse, the control participants generally displayed a faster rate of increase and a slower rate of deceleration rate than did the experimental participants. Third, compared with the control group, subjects in the experimental group consistently showed lower levels of problem behavior. Finally, a more encouraging finding is that the program seemed to produce sustained effects in decreasing the occurrence of adolescent risk behavior after the intervention. Students who participated in the program remained to score lower on a broad range of youth risk indicators than did the control students after the program had completed, which indicates that the Project PATHS has long-term effects in preventing problem behaviors among Hong Kong adolescents. Further analyses based on the experimental subjects who found the program to be beneficial to their development only showed similar results. These findings basically suggest that the Project PATHS is a strong protective factor for students joining the program, which delayed adolescents' involvement in risk behavior. 

As such, the present findings reinforced previous objective outcome evaluation findings based on both general linear models [15] and linear mixed methods [13, 14]. In conjunction with prior results based on objective outcome evaluation, subjective outcome evaluation, qualitative evaluation via focus groups, qualitative evaluation via diaries, process evaluation, and interim evaluation, the existing evaluation findings from the Project PATHS provide sound evidence that the program is an effective approach to youth risk behavior prevention, with sustained improvements in different problem behaviors. Shek and Yu [2] remarked that there is a dearth of effective positive youth development programs in different Chinese contexts. Obviously, based on the available research findings, the Project PATHS is a notable exception. Of course, if resources permit, further longitudinal data based on the participants of the study should be carried out. 

Researchers have long claimed [28] the importance of “building bridges between youth development and risk-prevention approaches in order to specify more clearly common predictors of multiple problem behaviors and how these are linked to healthy adjustment.” (page 90). More specifically, Bradshaw and Guerra proposed that one future direction for youth risk behavior research is to examine more fully the associations among context, youth competences, and risk behaviors across time and culture [28]. The present study may be considered a quick response. Apparently, following this study, a lot of meaningful work can be done in the future, such as the identification of core positive youth development constructs that directly contribute to the prevention of risk behaviors in Chinese adolescents; the examination of relative strength of each construct across different behaviors; the replication of the Project PATHS in different Chinese contexts. 

In conjunction with the previous research findings of the Project PATHS [29–31], existing evaluation findings basically suggest that the program is an effective one in promoting adolescent development and reducing the related risk behavior. Nevertheless, despite the positive findings of the study, it is noteworthy that there are several limitations of the study. First, as only two years of follow-up were involved in the program, only the short-term follow-up effect of the program was revealed. Obviously, it would be exciting to examine the program effect over a longer period of time. Second, although the schools did not differ in their background characteristics (when schools were used as units of analyses) and student characteristics, it would be helpful to examine the program effects in schools with different characteristics such as school banding. Third, as risk behavior was reported by adolescents alone, it would be helpful if data based on other informants such as parents and teachers could be collected in future studies. Despite these limitations, this study is very significant as it is the first known evaluation study in which longitudinal data were collected over such a long period of time in a Chinese context.

## Figures and Tables

**Table 1:** Number of collected questionnaires across waves.

*N* (School)	Wave 1	Wave 2	Wave 3	Wave 4	Wave 5	Wave 6	Wave 7	Wave 8
	48	47^a^	44^b^	44^c^	43	43	43	43
No. of participants	7,846	7,388	6,939	6,697	6,876	6,733	6548	6492
Control Group	3,797	3,654	3,765	3,698	3,757	3,727	3669	3640
Male	1,936	1,876	1,896	1,888	1,874	1,894	1,894	1,865
Female	1,613	1,619	1,666	1,599	1,682	1,679	1,689	1,716
Experimental Group	4,049	3,734	3,174	2,999	3,119	3,006	2,879	2,852
Male	2,154	1,998	1,691	1,548	1,632	1,591	1,536	1,533
Female	1,745	1,571	1,283	1,259	1,312	1,278	1,225	1,272
% of successfully matched	98%	96%	97%	98%	99%	97%	93%	91%

^a^1 Experimental school (*n* = 207) had withdrawn after Wave 1.

^b^3 Experimental schools (*n* = 629) had withdrawn after Wave 2.

^c^1 Experimental school (*n* = 71) had withdrawn after Wave 4.

**Table 2:** Internal consistency and mean inter-item correlations for composite problem behavior indicators.

	Wave 1		Wave 2		Wave 3		Wave 4		Wave 5		Wave 6		Wave 7		Wave 8	
	*α*	mean^a^	*α*	mean^a^	*α*	mean^a^	*α*	mean^a^	*α*	mean^a^	*α*	mean^a^	*α*	mean^a^	*α*	mean^a^
DELINQ	0.77	0.32	0.79	0.35	0.79	0.35	0.82	0.40	0.81	0.38	0.82	0.38	0.80	0.39	0.76	0.34
DRUG	0.76	0.56	0.81	0.58	0.77	0.56	0.82	0.61	0.79	0.59	0.83	0.63	0.78	0.60	0.72	0.58
BEINT	0.76	0.47	0.78	0.47	0.79	0.49	0.78	0.46	0.79	0.47	0.79	0.46	0.76	0.41	0.73	0.37

^a^Mean interitem correlation.

All parameters were significant (*P* < .05).

Note: DELINQ: delinquency; DRUG: substance abuse; BEINT: problem behavior intention.

**Table 3:** Growth curve models for problem behavior indicators with subjects joining the Tier 1 Program as experimental subjects.

	Dependent variables													
	DELINQ	DRUG	BEINT	IPS	LPS	Ketamine	Cannabis	Solvent	Ecstasy	Heroin	Sexual	Violence	Night	Trespasses
*Intercept*														
Initial status	.22**	.07**	1.19**	.00	.02**	.00	.00	.03**	.00	.00	.02*	.04**	.05**	.04**
Group	−.03**	−.01	−.02*	.00	−.01	.00	.00	−.01	.00	.00	.00	−.01	−.01	−.01
Gender	−.05**	−.01*	−.04**	.00	.00	.00	.00	.00	.00	.00	−.01*	−.03**	−.03**	−.01
Age	.06**	.04**	.06**	.01*	.01**	.00	.01**	.01	.01**	.00	.02**	.03**	.09**	.02**

*Linear*														
Initial status	.26**	.06**	.21**	.01**	.01**	.02**	.01**	.02**	.01**	.01**	.02**	.05**	.06	.02**
Group	−.03*	−.01**	.00	−.01*	−.01*	−.01*	−.01*	−.01*	−.01*	−.01*	−.005*	−.02*	−.03**	−.01**
Gender	−.01	.00	.01	.001*	−.01*	−.01*	−.01*	−.01*	−.01*	−.01**	−.004*	−.03**	−.02*	−.01*
Age	−.04**	−.01	−.01	−.01*	−.01**	−.01	−.01**	−.01*	−.01*	−.01*	.00	−.02**	−.02*	−.01*

*Quadratic*														
Initial status	−.08	−.01	−.06**	−.002**	−.003**	−.003**	−.002**	−.004**	−.003**	−.002**	—	−.01**	−.01	−.005**
Group	.01	.002*	−.01*	.002*	.001*	.00	.001*	.002*	.002*	.002*	—	.003*	.01**	.002*
Gender	−.01	.00	−.01*	.001*	.00	.00	.00	.00	.001*	.002*	—	.01**	.00	.00
Age	.01	.002*	.00	.00	.002*	.002*	.001*	.00	.00	.00	—	.005**	.003*	.003*

*Cubic*														
Initial status	.01	—	.01**	—	—	—	—	—	—	—	—	—	—	—
Group	.00	—	.002*	—	—	—	—	—	—	—	—	—	—	—
Gender	.00	—	.001*	—	—	—	—	—	—	—	—	—	—	—
Age	.00	—	.00	—	—	—	—	—	—	—	—	—	—	—

Note: DELINQ: scale score of the delinquency scale; DRUG: scale score of the substance abuse scale; BEINT: scale score of the intention of problem behavior scale; IPS: composite score of using illegal drugs (ketamine, cannabis, ecstasy and heroin); LPS: composite score of using legal drugs (organic solvent and cough medicine); Sexual: item score of having sexual behavior with others; Violence: item score of violent behavior; Night: item score of staying outside home overnight without parental approval; Trespasses: item score of trespasses.

*P < 0.05, **P < 0.01.

**Table 4:** Growth curve models for problem behavior indicators with subjects joining the Tier 1 Program and perceive the program as effective being experimental subjects.

	Dependent variables														
	DELINQ	DRUG	BEINT	IPS	LPS	Tobacco	Alcohol	CAS	Solvent	Ecstasy	Heroin	Damage	Violence	Night	Trespasses
*Intercept*															
Initial status	.22**	.07**	1.20	.00	.02**	.09**	.39**	.24**	.03**	.00	.00	.11**	.05**	.05**	.04**
Group	−.03**	−.01	−.01*	.00	.00	−.02*	−.03*	−.03**	.00	.00	.00	−01	−.01	−.01	−.01
Gender	−.05**	−.01**	−.05	.00	.00	−.03**	−.08**	−.05**	−.01	.00	.00	−.05**	−.03**	−.03**	−.01
Age	.06**	.04**	.06	.01**	.01**	.15**	.12**	.13**	.01*	.01**	.01	.05**	.03**	.09**	.02**

*Linear*															
Initial status	.25**	.07**	.22**	.01**	.01**	.22**	.40**	.31**	.02**	.02**	.01**	.12**	.05**	.08**	.02**
Group	−.02*	.00	.02	−.01*	−.01**	.04*	.04*	.04**	−.01**	−.01*	−.01**	−.04*	−.01*	−.02*	−.01**
Gender	−.01	.00	.02*	−.01**	−.01*	.04*	.09**	.06**	−.01*	−.01*	−.01**	−.02	−.03**	−.02**	−.01*
Age	−.03	−.01*	.00	.00	−.01*	..04*	.00	.02	−.01*	.00	.00	−.02	−.02**	−.01	−.01*

*Quadratic*															
Initial status	−.08**	−.01	−.06**	−.002*	−.003**	−.05**	-.10**	−.08**	−.004**	−.003**	−.002**	−.04**	−.01**	−.01	−.004**
Group	.01*	.00	−.02**	.001*	.001**	−.02*	−.02*	−.02**	.002**	.002*	.001**	.02**	.00	.003*	.003**
Gender	.00	.00	−.02**	.001*	.00	−.02**	−.04**	−.03**	.00	.001*	.001*	−.01	.01*	.00	.00
Age	.00	.00	.00	.00	.00	−.02*	.00	−.02*	.00	.00	.00	.00	.003*	.00	.003*

*Cubic*															
Initial status	.01**	—	.01**	—	—	.01**	.01**	.01**	—	—	—	.003*	—	—	—
Group	.00	—	.002**	—	—	.003*	.003*	.003**	—	—	—	−.003**	—	—	—
Gender	.00	—	.002*	—	—	.003*	.005**	.004**	—	—	—	.00	—	—	—
Age	.00	—	.00	—	—	.004**	.00	.003*	—	—	—	.00	—	—	—

Note: DELINQ: scale score of the delinquency scale; DRUG: scale score of the substance abuse scale; BEINT: scale score of the intention of problem behavior scale; IPS: composite score of using illegal drugs (ketamine, cannabis, ecstasy and heroin); LPS: composite score of using legal drugs (organic solvent and cough medicine); CAS: composite score of using tobacco and alcohol; Damage: item score of damaging other's property; Violence: item score of violent behavior; Night: item score of staying outside home overnight without parental approval; Trespasses: item score of trespasses.

*P < 0.05, **P < 0.01.
